# Oral Manifestations in Children and Adolescents with Eating Disorders or Eating-Disorder Risk: A Systematic Review and Stratified Meta-Analysis

**DOI:** 10.3390/children13101439

**Published:** 2026-10-20

**Authors:** Paula Luis-Gómez, David León-López, Isabel Crespo-Gallardo, Milagros Martín-Jiménez, Mª Carmen Jiménez-Sánchez, Victoria Areal-Quecuty, Juan J. Segura-Egea, María León-López

**Affiliations:** 1 Department of Stomatology, Endodontic Division, School of Dentistry, University of Sevilla, C/Avicena s/n, 41009 Sevilla, Spain; pauluigom@alum.us.es (P.L.-G.); icrespo@us.es (I.C.-G.); mmartin33@us.es (M.M.-J.); mjimenez39@us.es (M.C.J.-S.); vareal@us.es (V.A.-Q.); 2 Family and Community Medicine, Hospital Universitario Virgen del Rocío, Andalusian Health Service, 41013 Seville, Spain; david.leon.lopez.sspa@juntadeandalucia.es; 3 San Isidoro University Center, Pablo de Olavide University, 41013 Seville, Spain

**Keywords:** eating disorders, anorexia nervosa, bulimia nervosa, eating disorder risk, tooth wear, dental caries, salivary flow rate, adolescents, stratified meta-analysis, systematic review

## Abstract

**Highlights:**

**What are the main findings?**

- Erosive tooth wear was more frequent in adolescents with clinically diagnosed anorexia nervosa and in screening-positive cohorts than in their respective controls.
- Unstimulated salivary flow was reduced in clinically diagnosed anorexia nervosa, although evidence was limited to two studies from a single specialized center.
- Evidence regarding caries experience was inconsistent, particularly among screening-positive adolescents.

**What are the implications of the main findings?**

- Oral findings may provide dental practitioners with an opportunity to support recognition of possible eating disorders and interdisciplinary referral.
- The certainty of the available evidence is low to very low, and the findings should therefore be interpreted cautiously.

**Abstract:**

Background: Eating disorders (EDs), primarily anorexia nervosa, bulimia nervosa, and related risk behaviors, are complex biopsychosocial conditions frequently accompanied by stomatognathic alterations. Systemic malnutrition and chronic purging behaviors significantly impact oral tissues, giving dental practitioners a valuable opportunistic position for early recognition. Objective: We aimed to systematically evaluate and quantitatively synthesize stomatognathic manifestations in children and adolescents with clinically diagnosed eating disorders compared to those with screening-identified eating-disorder risk. Material and Methods: A systematic review and stratified meta-analysis were conducted according to PRISMA 2020 guidelines (PROSPERO: CRD420261490862). Comprehensive searches were executed across PubMed, Scopus, and Embase on 24 July 2026. Eligible primary studies evaluated oral outcomes in pediatric cohorts aged nine to eighteen years. Studies were stratified into clinical diagnoses and screening-defined at-risk cohorts, utilizing random-effects meta-analyses and GRADE assessments. Results: A total of twelve studies were included (nine comparative studies with healthy controls and three single-arm descriptive case series; seven comparative studies contributed to quantitative meta-analyses). In the stratified meta-analysis for erosive tooth wear (four studies, N = 1030), patients with clinically diagnosed anorexia nervosa exhibited an individual study odds ratio of 7.72 (95% CI: 2.24–26.64), whereas screening-positive community cohorts showed a pooled odds ratio of 3.50 (95% CI: 1.49–8.23; three studies); between-group differences remain descriptive. For caries experience (three studies, N = 392), clinically diagnosed patients showed elevated DMFT scores in a single study (MD = +1.90), whereas screening-identified school cohorts showed no statistically significant elevation (pooled MD = +0.88, 95% CI: −0.95 to 2.71), with high heterogeneity (I^2^ = 86.8%). Unstimulated whole salivary flow rate was significantly reduced in diagnosed clinical anorexia (two studies, N = 156, MD = −0.20 mL/min); however, both studies originated from a single specialized center in Poznan, Poland, precluding multicenter generalization. In nationwide survey data, adolescents exhibiting unhealthy weight control showed no statistically significant differences in poor subjective oral health. Conclusions: Stomatognathic manifestations in children and adolescents vary descriptively across clinical diagnostic and screening-risk cohorts, characterized primarily by erosive tooth wear and resting salivary hypofunction in diagnosed anorexia nervosa. Although the certainty of evidence remains very low across evaluated outcomes and erosive tooth wear is etiologically multifactorial rather than pathognomonic, dental practitioners occupy a valuable position for recognizing suspicious non-carious wear, prioritizing conservative preventive stabilization, and coordinating prompt interdisciplinary referral with pediatric medical and mental health teams.

## 1. Introduction

Eating Disorders (EDs) represent a major global public health challenge among children and adolescents, with epidemiological surveillance documenting notable increases in pediatric eating pathology over recent years, particularly during and following the COVID-19 pandemic [1]. In contemporary psychiatric nosology (DSM-5 and ICD-11), EDs encompass a spectrum of clinical conditions defined by persistent disturbances in eating or weight-control behaviors that alter nutritional intake and significantly impair physical health and psychosocial functioning [2]. While Anorexia Nervosa (AN) and Bulimia Nervosa (BN) represent the most extensively characterized entities in pediatric cohorts, the spectrum also encompasses Binge-Eating Disorder (BED), Avoidant/Restrictive Food Intake Disorder (ARFID), and Other Specified Feeding or Eating Disorders (OSFED), reflecting complex interactions among genetic susceptibility, neurochemical alterations, psychological vulnerability, and sociocultural pressures [1,2].

At the organ level, the oral cavity functions as an anatomical site frequently exhibiting physical sequelae of disordered eating [3]. In individuals engaging in self-induced vomiting or frequent regurgitation, recurrent exposure of dental enamel to gastric hydrochloric acid (pH 1.0–2.0) can lead to accelerated chemical dissolution of hard dental tissues, clinically described as erosive tooth wear (ETW) or perimolysis [3,4]. However, it is essential to emphasize that erosive wear is etiologically multifactorial and not pathognomonic of eating disorders; identical wear patterns may arise from gastroesophageal reflux disease (GERD), chronic rumination, frequent consumption of acidic dietary beverages, or acidic medications [4,5]. Additionally, severe nutritional restriction and chronic hypovolemia can impair salivary gland secretion, inducing glandular hypofunction and alterations in salivary biochemical composition [2,6].

These physical sequelae extend beyond biological damage, contributing to dentinal hypersensitivity, impaired masticatory function, altered esthetics, and deteriorated oral health-related quality of life (OHRQoL) [7]. Because adolescents with EDs often experience shame or ambivalence regarding their behaviors, covert purging and restrictive practices may remain concealed from family members and primary physicians for months or years. Under these circumstances, dentists and pediatric dental specialists may observe characteristic oral signs during routine clinical examinations, providing opportunities for supportive, non-stigmatizing medical referral [8].

Over the past two years, several comprehensive systematic reviews have evaluated oral manifestations of eating disorders across broad populations (Nijakowski et al., 2023; Valeriani et al., 2024; Chan et al., 2024) [4,9,10]. However, these previous syntheses predominantly focused on adult cohorts or combined adult, adolescent, and mixed community samples without evaluating whether stomatognathic effect sizes diverge between clinically confirmed psychiatric diagnoses and self-reported psychometric screening risk. Furthermore, the pediatric literature involves unique developmental considerations, including the transition through mixed dentition and shorter cumulative illness durations. The lower age boundary of 9 years was prespecified in our PROSPERO protocol (CRD420261490862), corresponding to the earliest documented pediatric clinical presentations of early-onset anorexia nervosa in the specialized literature [7,11,12]. Therefore, this systematic review and stratified meta-analysis was specifically designed to evaluate pediatric and adolescent populations, providing a dedicated stratified synthesis that evaluates oral effect estimates according to diagnostic certainty (clinically confirmed ED versus psychometric screening risk) with the literature updated to July 2026.

## 2. Materials and Methods

### 2.1. Study Design, Protocol and PECO Framework

This investigation was conducted as a systematic review and quantitative meta-analysis of the scientific literature in accordance with the Preferred Reporting Items for Systematic Reviews and Meta-Analyses (PRISMA 2020) statement [13]. The study protocol was registered on the International Prospective Register of Systematic Reviews (PROSPERO; registration number: CRD420261490862) to guide literature retrieval, study selection, critical appraisal, and statistical synthesis. No post hoc protocol amendments or deviations occurred during the review process.

The research question was formulated according to the PECO framework:

- Population (P): Children and adolescent patients aged 9 to 18 years.
- Exposure (E): Stratified into: (1) Clinically Diagnosed ED (confirmed clinical diagnosis of AN, BN, or related ED established via standardized psychiatric/pediatric criteria such as DSM-IV, DSM-5, or ICD-10), and (2) Screen-Positive/At-Risk Cohorts (adolescents identified as having high ED risk or disordered eating behaviors via validated psychometric screening instruments, such as EAT-26 score ≥ 20, BITE score ≥ 20/severity ≥ 10, or population health survey criteria).
- Comparison (C): Healthy young control subjects without eating disorder diagnoses or elevated risk scores on screening instruments.
- Outcomes (O): Primary outcomes comprised erosive tooth wear (prevalence and Odds Ratio) and salivary flow rate (mL/min, unstimulated and stimulated). Secondary outcomes included caries experience (DMFT/dmft index), soft-tissue alterations (cheilitis, mucositis, sialadenosis), and subjective oral health perceptions.

The overarching research question was: What are the stomatognathic manifestations and quantitative effect sizes in children and adolescents with clinically diagnosed eating disorders or screening-identified eating disorder risk compared to healthy controls?

### 2.2. Search Strategy and Information Sources

A comprehensive literature search was executed across three major international biomedical databases (PubMed/MEDLINE, Scopus, and Embase) on 24 July 2026. Search strategies incorporated Medical Subject Headings (MeSH) and EMTREE terms combined with free-text keywords related to eating disorders, adolescents, and stomatognathic manifestations.

- PubMed/MEDLINE: (“Feeding and Eating Disorders”[Mesh] OR “Anorexia Nervosa”[Mesh] OR “Bulimia Nervosa”[Mesh] OR “Binge-Eating Disorder”[Mesh] OR “Anorexia Nervosa”[tiab] OR “Bulimia Nervosa”[tiab] OR “Eating Disorder*”[tiab] OR “Disordered Eating”[tiab] OR “Eating Disorder Risk”[tiab] OR “Bulimic Symptom*”[tiab] OR “Purging”[tiab] OR “Self-induced vomiting”[tiab]) AND (“Tooth Wear”[Mesh] OR “Tooth Erosion”[Mesh] OR “Dental Caries”[Mesh] OR “Saliva”[Mesh] OR “Tooth Wear”[tiab] OR “Erosive Tooth Wear”[tiab] OR “Dental Erosion”[tiab] OR “Perimolysis”[tiab] OR “Dental Caries”[tiab] OR “DMFT”[tiab] OR “Salivary Flow”[tiab] OR “Sialadenosis”[tiab] OR “Cheilitis”[tiab]) AND (“Child”[Mesh] OR “Adolescent”[Mesh] OR “Pediatrics”[Mesh] OR “Child*”[tiab] OR “Adolescen*”[tiab] OR “Teen*”[tiab] OR “Youth”[tiab] OR “Pediatric*”[tiab]).
- Scopus: TITLE-ABS-KEY((“Eating disorder*” OR “Anorexia” OR “Bulimia” OR “Disordered eating” OR “Eating disorder risk” OR “Purging” OR “Binge eating”) AND (“Tooth wear” OR “Dental erosion” OR “Erosive tooth wear” OR “Perimolysis” OR “Dental caries” OR “DMFT” OR “Salivary flow” OR “Sialadenosis”) AND (“Adolescent*” OR “Child*” OR “Teen*” OR “Youth” OR “Pediatric*”).
- Embase: (‘eating disorder’/exp OR ‘anorexia nervosa’/exp OR ‘bulimia’/exp OR ‘disordered eating’:ti,ab OR ‘eating disorder risk’:ti,ab OR ‘purging’:ti,ab) AND (‘tooth wear’/exp OR ‘dental erosion’/exp OR ‘dental caries’/exp OR ‘saliva’/exp OR ‘perimolysis’:ti,ab OR ‘erosive tooth wear’:ti,ab) AND (‘child’/exp OR ‘adolescent’/exp OR ‘pediatric’:ti,ab).

In addition, reference lists of included primary studies and relevant previous systematic reviews were hand-searched to identify potentially eligible investigations not captured by electronic queries.

### 2.3. Eligibility Criteria and Cohort Screening Protocol

Inclusion criteria comprised: (1) primary studies (controlled cross-sectional, case–control, cohort, and single-arm descriptive case series) evaluating children and adolescents aged 9 to 18 years; (2) exposure defined as either clinically diagnosed ED (DSM/ICD criteria) or screening-identified high ED risk (validated psychometric instruments); (3) primary outcomes requiring direct, objective clinical or laboratory examinations of dental, mucosal, or salivary parameters; and (4) for prespecified secondary outcomes evaluating patient-reported oral symptoms and subjective oral health perceptions, large-scale validated psychometric surveys were eligible for qualitative synthesis but strictly excluded from objective quantitative meta-analyses.

### 2.4. Study Selection, Data Extraction and Risk of Bias Assessment

Literature screening and study selection were executed independently by two reviewers (P.L.-G. and D.L.-L.) using reference management software (Mendeley v2.149.0). Following automated and manual deduplication, titles and abstracts were screened against predefined eligibility criteria. Potentially eligible reports were retrieved for full-text assessment. Any discrepancies during the screening and selection phases were resolved through consensus discussion involving senior investigators (J.J.S.-E. and M.L.-L.).

Data extraction was conducted independently by two reviewers (P.L.-G. and D.L.-L.) using a standardized data extraction template. Extracted variables comprised: first author and year of publication, country and study design, pediatric cohort characteristics (sample sizes, age ranges, and diagnostic criteria for clinical ED or psychometric screening instruments), diagnostic classification, objective stomatognathic outcome measures (erosive tooth wear indices, DMFT/dmft scores, resting and stimulated salivary flow rates, and periodontal/mucosal signs), raw quantitative data (means, standard deviations, event counts, and group totals), and main clinical conclusions. When required data or standard deviations were missing or ambiguously reported, corresponding study investigators were contacted for clarification.

Methodological quality and risk of bias were independently evaluated by two reviewers (P.L.-G. and M.L.-L.) using design-specific Joanna Briggs Institute (JBI) Critical Appraisal Checklists. Overall risk of bias was categorized according to prespecified domain-based decision rules: Low Risk (≥70% affirmative criteria with no critical domain compromised), Moderate Risk/Some Concerns (50–69% affirmative criteria or residual unadjusted confounding regarding dietary acid intake or socioeconomic background), and High Risk (<50% affirmative criteria, or absence of direct clinical dental examination). For descriptive case series, overall risk-of-bias judgments were based exclusively on the applicable domains of the JBI Critical Appraisal Checklist for Case Series. The absence of an unexposed comparator was considered an inherent feature of the study design and was not, by itself, used as a criterion for downgrading the overall risk-of-bias judgment.

Corresponding authors of four studies were contacted via email to clarify missing variance parameters or confirm volumetric measurement units (e.g., salivary collection protocols in Paszynska et al. [14]). Clarifications regarding standardized resting flow units were confirmed, whereas no additional unpublished datasets modifying reported event counts were obtained. Separately, attempts were made to contact authors of the eight reports for which full texts could not be retrieved, with the purpose of obtaining the complete articles; no additional full texts were obtained through these contacts within the four-week response window. The two contributing salivary cohorts in Paszynska et al. (2015, 2018) [14,15] represented chronologically independent patient samples recruited consecutively across non-overlapping timeframes (2011–2013 for the 2015 study; 2014–2016 for the 2018 study), ruling out double-counting of individual participants.

### 2.5. Statistical Synthesis, Quantitative Meta-Analysis, and GRADE Evidence Assessment

Quantitative meta-analyses, leave-one-out sensitivity analyses, and publication-grade forest plots were performed using JASP (version 0.18.3, JASP Team, University of Amsterdam, Netherlands), executing the metafor package (version 4.4-0) within its integrated R computing architecture (R version 4.3.2) via Restricted Maximum Likelihood (REML) estimation. For binary outcomes (erosive tooth wear), raw 2 × 2 contingency data were systematically extracted to recalculate unadjusted Odds Ratios (OR) and 95% Confidence Intervals within a uniform random-effects framework, ensuring mathematical consistency across studies with differing multivariable adjustment models; published multivariable-adjusted estimates were extracted and synthesized qualitatively. For continuous outcomes measured on identical clinical scales across studies (DMFT index and salivary flow rate in mL/min), the effect size was calculated as the raw Mean Difference (MD) with corresponding 95% Confidence Intervals using inverse-variance random-effects weighting.

A random-effects model using the Restricted Maximum Likelihood (REML) estimator was specified a priori. Because meta-regression is generally considered unreliable and underpowered with fewer than 10 studies per covariate (Cochrane Handbook), no meta-regression was performed; clinical and exposure heterogeneity were instead systematically addressed through pre-specified stratified subgroup meta-analyses: Subgroup 1 (Clinically Diagnosed ED) and Subgroup 2 (Screening-Positive/At-Risk Cohorts). To avoid ecological fallacy, the overall unstratified pooled estimates are presented strictly as secondary heuristic overviews, while the stratified subgroups represent the primary clinically interpretable findings. Leave-one-out sensitivity analyses were systematically executed across all pooled outcomes to test the stability of effect estimates.

Statistical heterogeneity across primary studies was evaluated using Cochran’s Q test (with significance set at *p* < 0.10) and quantified using the I^2^ statistic, categorized as low (<25%), moderate (25–50%), substantial (50–75%), or high (>75%), alongside reporting of τ^2^. In accordance with Cochrane recommendations, formal statistical testing for small-study effects and publication bias (e.g., Egger’s regression asymmetry test) was omitted due to low statistical power when fewer than 10 studies were available per outcome.

The overall certainty of evidence across meta-analyzed and synthesized stomatognathic outcomes was systematically evaluated using the Grading of Recommendations Assessment, Development, and Evaluation (GRADE) framework. Certainty was classified as High, Moderate, Low, or Very Low across five standard domains: risk of bias, inconsistency, indirectness, imprecision, and publication bias.

## 3. Results

### 3.1. Study Selection and PRISMA Flow Dynamics

**Figure 1:**
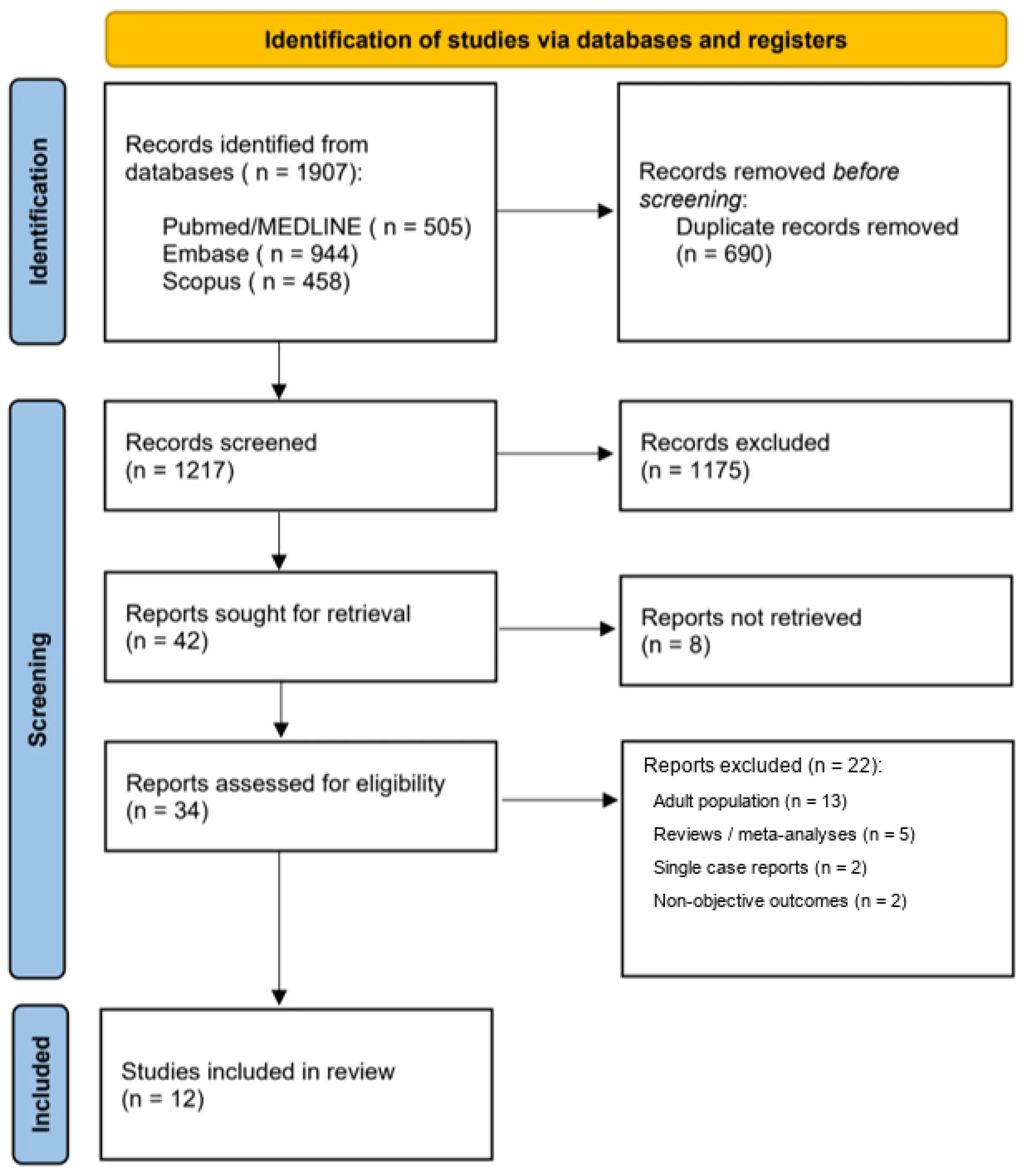
PRISMA 2020 flow diagram depicting database searches, deduplication, screening, eligibility, and study inclusions.

The electronic database search retrieved 1907 citations (PubMed: 505, Scopus: 458, Embase: 944). Following automated and manual deduplication, 690 duplicate records were removed, leaving 1217 unique records for title and abstract screening. Of these, 1175 records were excluded based on predefined criteria: adult-only populations (*n* = 642), animal or in vitro experimental models (*n* = 184), absence of stomatognathic or dental outcomes (*n* = 219), and narrative reviews, commentaries, or case reports lacking primary comparative data (*n* = 130). Full-text assessment was sought for 42 articles, of which 8 could not be retrieved after extensive institutional interlibrary loan requests and direct author contact (archival local journals lacking digitized repositories). Among the 34 full-text articles assessed, 22 were excluded with documented reasons: adult cohorts (*n* = 13), narrative reviews or meta-analyses without primary empirical pediatric data (*n* = 5), single case reports (*n* = 2), and absence of objective stomatognathic clinical outcomes (*n* = 2) (Table 1). Ultimately, 12 primary observational studies met all inclusion criteria and were incorporated into the systematic review (Figure 1).

### 3.2. Methodological and Quantitative Characteristics of Included Studies

Table 2 synthesizes the demographic, methodological, clinical, and raw quantitative meta-analytic data extracted from the 12 included studies.

### 3.3. Qualitative Synthesis of Stomatognathic Manifestations

#### 3.3.1. Hard Tissue Pathology: Dental Erosion and Caries Susceptibility

Enamel dissolution secondary to acidic erosive tooth wear (ETW) represented the most frequently examined hard tissue alteration. In clinically diagnosed adolescent Anorexia Nervosa inpatients, Paszynska et al. (2022) [38] observed erosive tooth wear (BEWE score ≥ 2) in 18.8% of patients (22/117) compared to 2.9% of healthy controls (3/103; raw OR = 7.72, 95% CI: 2.24–26.64). In population-based screening studies, Hermont et al. (2013) [35] identified severe bulimic risk behavior using the BITE questionnaire, reporting an unadjusted OR of 8.53 (95% CI: 2.64–27.59) and an adjusted OR of 10.04 (95% CI: 2.44–41.34) for tooth wear. Similarly, Ximenes et al. (2010) [42] identified high ED risk via EAT-26/BITE, finding wear in 13.5% of at-risk youth (29/215) versus 6.0% of controls (26/435; raw OR = 2.45, 95% CI: 1.41–4.28). Conversely, Brandt et al. (2017) [40] evaluated high-severity bulimic risk behavior via BITE, finding wear in 16.7% of at-risk girls (2/12) versus 8.3% of controls (4/48); this association was not statistically significant (Fisher’s exact test *p* = 0.590; OR = 2.20, 95% CI: 0.35–13.73), demonstrating that screening-identified risk does not uniformly translate into detectable clinical wear in small adolescent samples.

#### 3.3.2. Salivary Physiology and Biochemical Alterations

Salivary physiology was evaluated in adolescent Anorexia Nervosa cohorts by Paszynska and colleagues (2015, 2017, 2018) [6,14,15]. In resting whole saliva, unstimulated flow rates were significantly lower in AN patients (0.28 ± 0.14 mL/min) than in matched controls (0.45 ± 0.18 mL/min; *p* < 0.001) [14], a finding replicated in their 2018 cohort (0.25 ± 0.12 vs. 0.46 ± 0.16 mL/min; *p* < 0.001) [15]. Stimulated parotid secretion was also markedly reduced (0.21 ± 0.11 vs. 0.48 ± 0.19 mL/min; *p* < 0.001) [6]. In addition to volumetric reduction, biochemical alterations were noted: parotid lysozyme concentration and total output were decreased, while salivary vaspin was elevated in AN, suggested by the authors as a potential neuroendocrine adaptation to starvation warranting further longitudinal investigation [6,15].

#### 3.3.3. Soft Tissue, Mucosal and Periodontal Inflammatory Signs

Ximenes et al. (2010) [42] documented significant associations between ED symptoms and oral mucositis (8.4%), angular cheilitis (7.0%), and sialadenosis (11.2%). Lesar et al. (2022) [43] demonstrated that marginal gingivitis (54.0%) and pharyngeal erythema (42.0%) predominate during initial disease phases, whereas friction tongue keratosis and scalloped tongue characterize chronic illness. Paszynska et al. (2022) [38] documented significantly higher gingival bleeding on probing in adolescent AN patients compared to controls (BOP: 20.0 ± 20.1% vs. 3.9 ± 8.1% of sites, *p* < 0.001), reflecting plaque-induced marginal gingival inflammation rather than loss of periodontal attachment.

#### 3.3.4. Subjective Oral Health and Weight Control Behaviors

In a nationwide web-based survey of 46,146 Korean adolescents, Jung and Jun (2022) [39] investigated the relationship between weight loss practices and subjective oral health. While crude descriptive percentages showed unadjusted complaints of oral symptoms among youth practicing unhealthy weight control methods (including fasting, diet pills, or purging), multivariable logistic regression adjusted for sociodemographic factors demonstrated no statistically significant elevation in self-reported toothache (adjusted OR = 1.14, 95% CI: 0.96–1.36, *p* = 0.141) or self-reported gingival bleeding (adjusted OR = 0.93, 95% CI: 0.75–1.15, *p* = 0.507). Similarly, the multivariable association with overall poor self-perceived oral health was not statistically significant (adjusted OR = 0.89, 95% CI: 0.71–1.12, *p* = 0.32). These findings demonstrate that self-reported oral health perceptions in youth engaging in disordered weight control behaviors do not reliably reflect physical disease burden, emphasizing the need for direct clinical examinations rather than reliance on subjective questionnaires.

### 3.4. Methodological Quality and Risk of Bias Appraisal

Methodological quality across the 12 included primary studies was evaluated across specific JBI domains (Table 3). Overall, 4 studies demonstrated low risk of bias, 7 exhibited moderate risk of bias (some concerns), and 1 showed high risk of bias. The most frequently compromised domain was confounding control: several cross-sectional school surveys did not adequately adjust for dietary acid intake, fluoride exposure, or socioeconomic status [15,19,20]. In addition, clinical spectrum restriction and inpatient setting divergence contributed to some concerns in Paszynska et al. (2022) [38]. Furthermore, studies by Hermont et al. (2021) [36], Montecchi et al. (2003) [41], and Lesar et al. (2022) [43] lacked a comparative control group, functioning strictly as single-arm descriptive case series. In screening studies, exposure misclassification represented an inherent vulnerability, as self-report psychometric scales (EAT-26, BITE) may identify non-clinical dieting behaviors alongside true subclinical pathology.

### 3.5. Quantitative Meta-Analysis Results

Quantitative meta-analyses were executed using a priori specified random-effects models with between-study variance estimated via Restricted Maximum Likelihood (REML). Data pooling was performed across three distinct clinical outcome domains.

#### 3.5.1. Erosive Tooth Wear (ETW)

**Figure 2:**
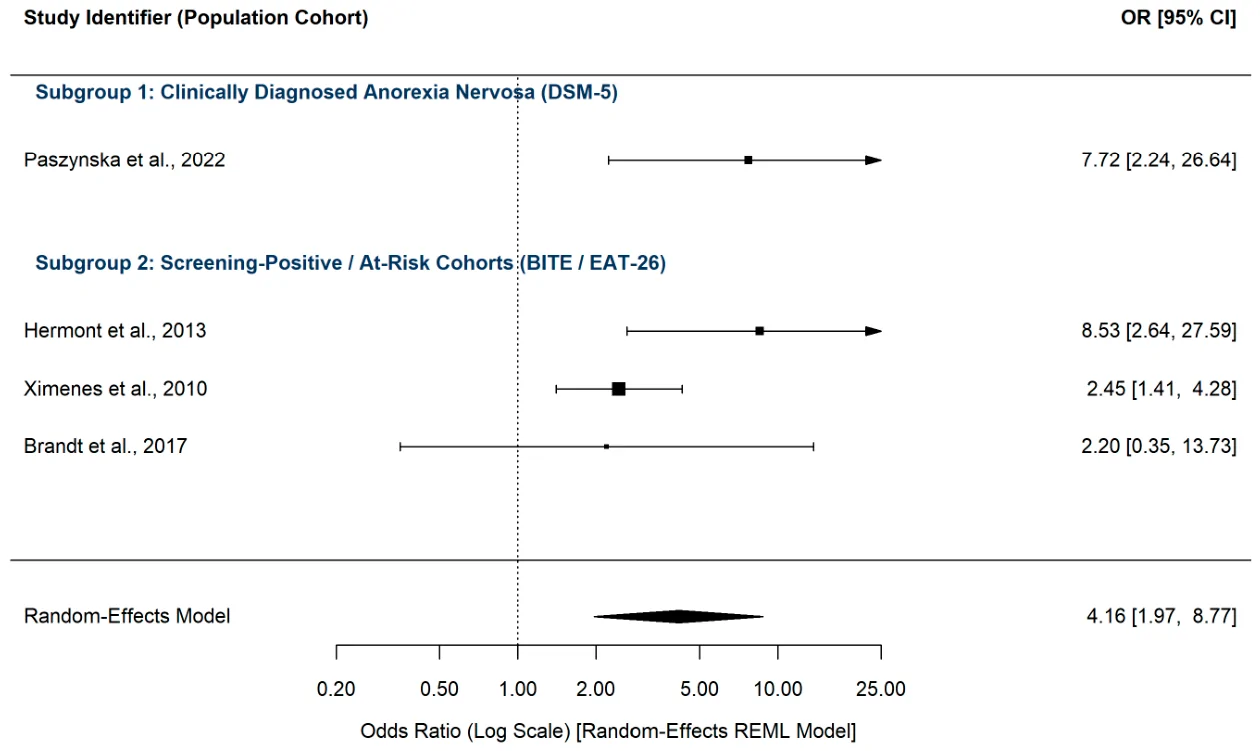
Forest plot of random-effects meta-analysis for erosive tooth wear (Odds Ratio) in children and adolescents stratified by diagnostic status (Subgroup 1: Clinically Diagnosed Anorexia Nervosa; Subgroup 2: Screening-Positive / At-Risk Cohorts). The black squares represent the individual study point estimates (Odds Ratios), with the size of the square proportional to the study weight; horizontal lines (and arrows) denote the corresponding 95% confidence intervals (CIs); the vertical dashed line indicates the line of no effect (OR = 1.0); and the black diamonds represent the pooled summary effect estimates and their 95% CIs.

Quantitative synthesis across four observational studies (Hermont et al., 2013 [35]; Brandt et al., 2017 [40]; Paszynska et al., 2022 [38]; Ximenes et al., 2010 [42]) evaluated erosive tooth wear in 1030 total participants (364 cases and 666 controls). When stratified by exposure category, the single study evaluating clinically diagnosed adolescent Anorexia Nervosa inpatients (Paszynska et al., 2022 [38]) reported an individual study OR of 7.72 (95% CI: 2.24–26.64), whereas quantitative synthesis across the three screening-positive/at-risk cohorts (Hermont et al., 2013 [35]; Brandt et al., 2017 [40]; Ximenes et al., 2010 [42]) yielded a pooled OR of 3.50 (95% CI: 1.49–8.23, *p* = 0.0041, I^2^ = 46.7%; Figure 2). Between-subgroup comparisons remain descriptive in the absence of formal subgroup difference testing. For exploratory heuristic completeness, the unstratified random-effects model yielded a summary OR of 4.16 (95% CI: 1.97–8.77, z = 3.74, *p* = 0.0002, I^2^ = 46.5%, τ^2^ = 0.2641). Leave-one-out sensitivity analysis confirmed the robustness of the overall association, with pooled ORs ranging from 3.26 (95% CI: 1.53–6.94) when omitting Hermont et al. [35] to 6.45 (95% CI: 2.98–13.96) when omitting Ximenes et al. [42], with all iterations retaining statistical significance (*p* < 0.005).

#### 3.5.2. Dental Caries Experience (DMFT Index)

**Figure 3:**
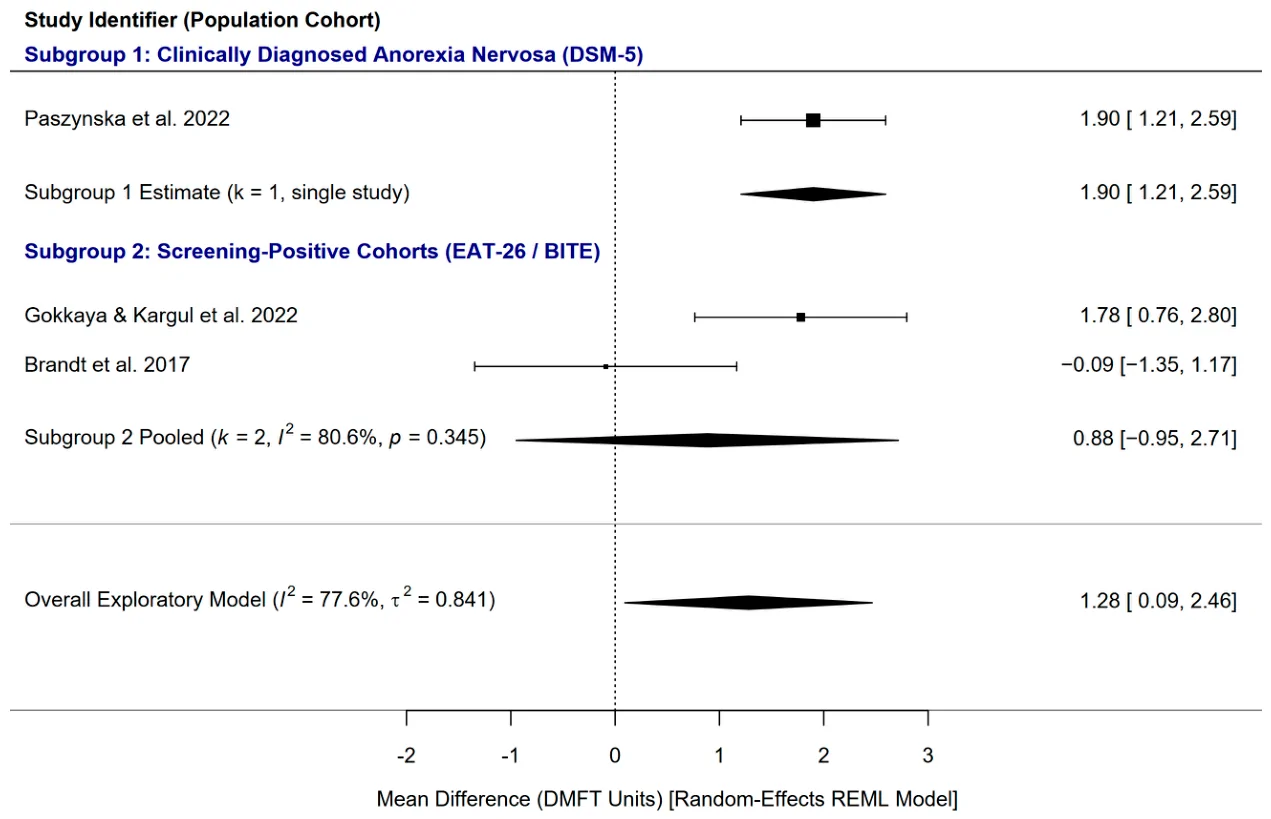
Forest plot of random-effects meta-analysis for DMFT caries index (Mean Difference) in children and adolescents stratified by diagnostic status (Subgroup 1: Clinically Diagnosed Anorexia Nervosa; Subgroup 2: Screening-Positive Cohorts).

Meta-analysis of caries experience across three studies reporting continuous DMFT scores (Paszynska et al., 2022 [38]; Gokkaya and Kargul, 2022 [37]; Brandt et al., 2017 [40]) evaluated 392 participants (166 cases and 226 controls). For caries experience, the clinically diagnosed AN subgroup was represented by a single study reporting an elevation in caries score (Paszynska et al., 2022 [38]: MD = +1.90, 95% CI: 0.99–2.81), whereas synthesis of the two screening cohorts showed no statistically significant elevation (pooled MD = +0.88, 95% CI: −0.95 to 2.71) accompanied by substantial statistical heterogeneity (I^2^ = 86.8%; Figure 3). These descriptive variations preclude concluding that caries experience follows a distinct diagnostic pattern across pediatric populations. For exploratory heuristic completeness, the unstratified model yielded a pooled MD of +1.26 (95% CI: 0.07–2.45, z = 2.08, *p* = 0.0378, I^2^ = 74.4%, τ^2^ = 0.8143). In leave-one-out sensitivity analysis, omitting Paszynska et al. (2022) [38] resulted in the loss of statistical significance for the overall caries estimate (pooled MD = +0.88, 95% CI: −0.95 to 2.71, *p* = 0.345), demonstrating the marked statistical fragility of the unstratified caries estimate.

#### 3.5.3. Unstimulated Whole Salivary Flow Rate

**Figure 4:**
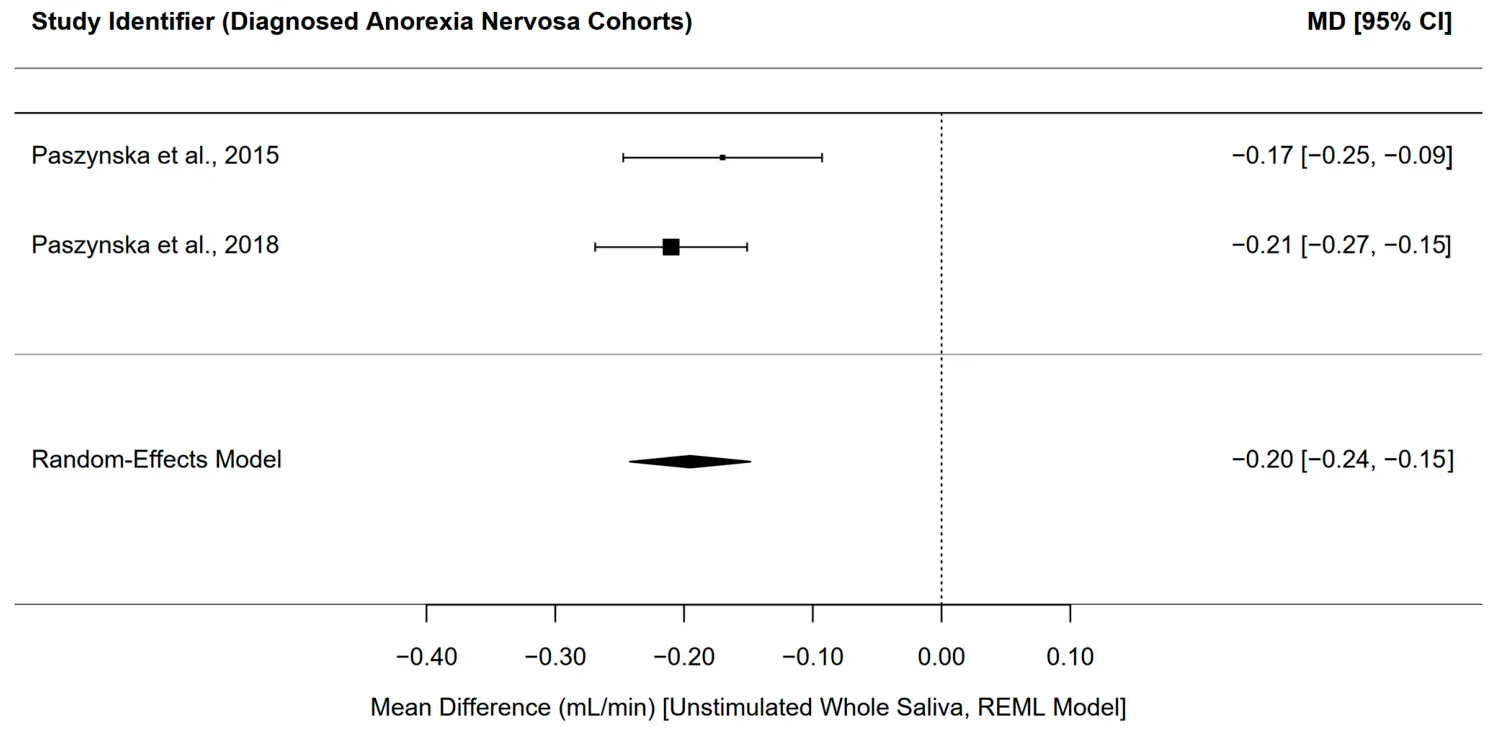
Forest plot of random-effects meta-analysis for unstimulated whole salivary flow rate (Mean Difference in mL/min) in adolescent Anorexia Nervosa cohorts.

Salivary flow rate parameters were analyzed strictly according to glandular collection protocols. Quantitative synthesis of resting, unstimulated whole saliva across two controlled observational case–control studies in diagnosed adolescent Anorexia Nervosa (Paszynska et al., 2015 [14]; Paszynska et al., 2018 [15]) included 156 participants (58 AN cases and 98 healthy controls). The two contributing cohorts represented chronologically independent patient samples recruited consecutively across non-overlapping timeframes (2011–2013 for the 2015 study; 2014–2016 for the 2018 study), ruling out participant double-counting. A random-effects model (REML) demonstrated a statistically significant reduction in resting salivary flow among AN patients (pooled MD = −0.20 mL/min, 95% CI: −0.24 to −0.15, z = −8.16, *p* < 0.0001; Figure 4). Statistical heterogeneity was calculated as negligible (Q = 0.65, df = 1, *p* = 0.4199, I^2^ = 0.0%, τ^2^ = 0.0000). We explicitly emphasize that with only two studies (k = 2), the statistical power to detect true between-study heterogeneity is virtually non-existent, and the reported I^2^ = 0.0% should not be misconstrued as mathematical proof of true homogeneity. Rather, pooling these cohorts serves as a quantitative synthesis of the only two existing controlled pediatric cohorts in the international literature evaluating resting flow in diagnosed Anorexia Nervosa under standardized volumetric protocols. Furthermore, because both investigations originated from the same specialized research center in Poznan, Poland, external multicenter validity remains to be established.

#### 3.5.4. Publication Bias and Small-Study Effects

In accordance with standard Cochrane and PRISMA guidance, formal statistical testing for publication bias (e.g., Egger’s regression or Begg’s rank correlation) could not be reliably performed because the number of studies in each meta-analytic synthesis was fewer than 10 (k = 4 for erosion, k = 3 for DMFT, k = 2 for saliva). Consequently, publication bias was deemed not assessable across all quantitative domains.

### 3.6. GRADE Summary of Findings and Evidence Certainty

The certainty of evidence for primary stomatognathic outcomes was systematically appraised separately for each exposure subgroup using the GRADE framework (Table 4). For erosive tooth wear (ETW), certainty was rated as VERY LOW (⊕◯◯◯) in clinically diagnosed AN. Observational evidence started at LOW certainty and was further downgraded for serious imprecision because the estimate was derived from a single study with a wide confidence interval; no upgrading criterion was applicable. Certainty was also rated as VERY LOW (⊕◯◯◯) in screening-positive cohorts due to residual confounding, indirectness associated with screening-defined rather than clinically confirmed ED exposure, and imprecision arising from the limited number of contributing studies. For caries experience (DMFT), certainty was rated as VERY LOW (⊕◯◯◯) in clinically diagnosed AN. Observational evidence started at LOW certainty and was further downgraded for serious imprecision because the estimate was based on a single study; no upgrading criterion was applicable. Certainty was also rated as VERY LOW (⊕◯◯◯) in screening-positive cohorts due to substantial inconsistency (I^2^ = 86.8%), residual confounding, indirectness associated with screening-defined exposure, and imprecision. For unstimulated whole salivary flow rate in clinically diagnosed AN, certainty was rated as VERY LOW (⊕◯◯◯), downgraded due to serious indirectness related to the single-center origin of both studies (Poznan, Poland), limiting external generalizability, and very serious imprecision arising from the small total sample size (N = 156) across only two studies (k = 2). For subjective oral health in screening-positive adolescents, data from a single nationwide survey (Jung and Jun, 2022) showed a non-significant association (OR = 0.89, 95% CI: 0.71–1.12), with VERY LOW (⊕◯◯◯) certainty due to risk of bias, indirectness, and imprecision.

## 4. Discussion

The findings of this systematic review and stratified meta-analysis suggest that children and adolescents with eating disorders and disordered eating risk exhibit distinct stomatognathic alterations, characterized primarily by increased odds of erosive tooth wear and salivary hypofunction in anorexia nervosa. A central methodological contribution of this review is the separate evaluation of clinically diagnosed patients and screening-positive adolescents, which avoids conflating clinically distinct exposure groups and allows their stomatognathic findings to be interpreted independently. Although numerical differences in effect estimates were observed between these populations, the clinically diagnosed AN subgroup was represented by a single study and no formal test for subgroup differences was performed; therefore, these differences should be interpreted descriptively and should not be considered evidence of a diagnostic severity gradient.

### 4.1. Erosive Tooth Wear: Multifactorial Etiology and Diagnostic Vigilance

Descriptively, the odds of erosive tooth wear observed in the single available inpatient Anorexia Nervosa cohort (OR = 7.72) were higher than the pooled estimate observed across community screening-positive cohorts (pooled OR = 3.50). However, because the clinical AN subgroup is represented by a single investigation, this observation must be interpreted descriptively and cannot be considered a statistically proven subgroup divergence. Gastric hydrochloric acid (pH 1.0–2.0) markedly undersaturates the oral fluid with respect to all tooth mineral phases. Unlike biofilm-mediated caries where demineralization thresholds are traditionally cited around pH 5.5 for hydroxyapatite and pH 4.5 for fluorapatite, erosive tooth wear involves direct surface dissolution that depends dynamically on titratable acidity, mineral saturation degree, salivary calcium and phosphate concentrations, salivary buffering capacity, and the protective properties of the acquired enamel pellicle [3,4,44]. Furthermore, attributing palatal enamel dissolution exclusively to eating disorders is biologically inaccurate. Dental erosion is multifactorial and can be induced or exacerbated by gastroesophageal reflux disease, chronic rumination, frequent consumption of acidic dietary beverages (sports drinks, carbonated sodas), and acidic medications [4,5,45]. Therefore, while oral wear patterns represent an important clinical flag, they cannot be considered pathognomonic of an eating disorder and must be contextualized within a comprehensive medical and behavioral history.

Direct comparison of our findings with previous systematic reviews and meta-analyses in adult and mixed-age populations (Nijakowski et al., 2023; Valeriani et al., 2024; Chan et al., 2024) [4,9,10] reveals essential developmental distinctions. In adult cohorts, pooled odds ratios for erosive tooth wear are consistently elevated to dramatic levels, frequently ranging from 5.6 to over 12.0 (e.g., Valeriani et al. reported OR = 5.61 in bulimia; Chan et al. reported OR = 10.96 in purging cohorts; Nijakowski et al. documented OR = 12.4 in eating disorder populations) [4,9,10]. In contrast, our stratified synthesis in pediatric community samples demonstrated a more moderate pooled odds ratio of 3.50 (95% CI: 1.49–8.23), while only the single specialized inpatient cohort of severe adolescent anorexia nervosa approached adult-like magnitude (OR = 7.72). The numerical differences observed between pediatric screening cohorts and previously reported adult clinical populations might partly reflect differences in cumulative disease duration, dentition stage, or exposure severity. However, these explanations remain hypotheses and were not directly evaluated in the included studies. Similarly, whereas adult meta-analyses frequently document marked elevations in DMFT scores driven by decades of binge eating, frequent exposure to fermentable carbohydrates, and chronic salivary hypofunction, our pediatric synthesis demonstrated that caries indices are not significantly elevated in community-screened adolescents (pooled MD = +0.88, 95% CI: −0.95 to 2.71). These contrasts indicate that oral disease burden in pediatric eating pathology is characterized by shorter cumulative illness chronicity and mixed dentition dynamics, underscoring that adult meta-analytic estimates cannot be uncritically extrapolated to pediatric practice. Crucially, a fundamental pathophysiological distinction must be recognized between behavioral phenotypes: dental erosion is mediated predominantly by direct chemical dissolution from regurgitated gastric acid in chronic purging behaviors, whereas in restrictive anorexia nervosa, oral risk is mediated primarily by severe nutritional deprivation and resting salivary hypofunction. In the included literature, detailed characterization of eating disorder subtypes and purging behaviors was available only in specialized clinical cohorts (Paszynska et al., 2015 [14], 2017 [6], 2018 [15], 2022 [38] restricted strictly to adolescent restrictive anorexia nervosa [AN-R]; Montecchi et al., 2003 [41] evaluated 49 restrictive and 31 binge-eating/purging anorexia patients, observing sialadenosis exclusively in vomiting patients; and Lesar et al., 2022 [43] described a mixed inpatient cohort comprising 27 AN, 6 BN, and 17 EDNOS patients), whereas school screening tools (BITE, EAT-26) capture broad attitudes without clinical differentiation. Importantly, our systematic review identified a complete absence of controlled studies evaluating clinically confirmed Bulimia Nervosa in strictly pediatric cohorts (<18 years), highlighting a profound gap in pediatric psychiatric dentistry.

### 4.2. Salivary Hypofunction in Adolescent Anorexia Nervosa

In contrast to direct chemical dissolution, salivary hypofunction in AN may reflect systemic consequences of severe malnutrition, including dehydration, electrolyte disturbances, and altered autonomic and metabolic regulation. Our meta-analysis of resting saliva confirmed a statistically significant reduction of −0.20 mL/min in adolescent AN patients compared to controls. Reduced salivary volume diminishes clearance of organic acids, compromises buffering capacity, and lowers salivary protein defenses, increasing vulnerability of both enamel and mucosal tissues. Crucially, methodological safeguards in both original protocols mitigate potential pharmacological confounding: both studies explicitly excluded participants receiving medications affecting salivary flow (Paszynska et al., 2015, 2018) [14,15], and collections were executed during the acute stage within the first week of hospitalization prior to the subsequent introduction of psychotropic pharmacotherapy in the minority (<20%) who required it. Collections were standardized to morning hours (9:00–11:00 AM) following a 60 min fast. Nonetheless, because the meta-analysis of resting saliva is based on only two studies (k = 2, N = 156) originating from the same specialized research program in Poznan, Poland, this pooled estimate cannot be interpreted as independent multicenter replication across diverse populations, and the calculated I^2^ = 0.0% has virtually no statistical power to rule out between-study heterogeneity. Further independent multicenter investigations are required to establish the universality of these salivary alterations.

### 4.3. Caries Heterogeneity and the Fragility of Caries Risk

These disparate findings suggest that caries susceptibility is not uniform across disordered eating phenotypes. Potential explanations—such as dietary carbohydrate restriction in restrictive anorexia versus salivary hypofunction or concurrent xerogenic psychotropic medications in severe chronic illness—represent biologically plausible hypotheses that warrant direct testing in prospective studies, as they were not systematically evaluated across the included primary literature. In school-based screening samples, individuals with disordered eating may maintain adequate tooth cleaning or compensate with restricted sugar intake, whereas in chronic severe anorexia, prolonged salivary hypofunction, starvation diets, or concurrent xerogenic medications may accelerate carious destruction. Caries risk in youth with EDs must therefore be evaluated on an individualized basis.

### 4.4. Subjective Perceptions and Clinical Management Considerations

In nationwide survey data from Jung and Jun (2022) [39], adolescents engaging in unhealthy weight-loss behaviors did not exhibit statistically significant odds of reporting poor subjective oral health (adjusted OR = 0.89, 95% CI: 0.71–1.12), self-reported toothache (adjusted OR = 1.14, 95% CI: 0.96–1.36), or gingival bleeding (adjusted OR = 0.93, 95% CI: 0.75–1.15). While this indicates that subjective health questionnaires may not capture the full extent of physical disease, direct inferences regarding a disconnect with clinical status remain limited by the absence of concurrent clinical dental examinations. Because preventive interventions were not empirically evaluated in the observational studies included in this review, clinical management strategies must be drawn from established pediatric dental consensus guidelines and the clinical erosion literature [2,4,7,8,26,44,45]. These professional consensus recommendations emphasize: (1) following acute acidic regurgitation or self-induced vomiting episodes, immediate vigorous toothbrushing with abrasive pastes should be avoided to prevent accelerated mechanical wear of acid-softened enamel; instead, immediate rinsing with water, sodium bicarbonate solution, or neutral sodium fluoride mouthrinses facilitates clearance and neutralizes intraoral acidity, whereas mechanical brushing should be delayed [4,26,44,45]; (2) daily home use of high-fluoride dentifrices (e.g., 5000 ppm F^−^ in adolescents with established permanent dentition) and professional application of topical fluoride varnishes or casein phosphopeptide-amorphous calcium phosphate (CPP-ACP) provide biochemical remineralization support to enhance acid resistance [2,7,44]; and (3) non-acidic salivary substitutes or sugar-free polyol chewing gums (xylitol) may alleviate xerostomic discomfort and stimulate protective salivary buffering.

In clinical practice, dental practitioners occupy an essential sentinel position for detecting subtle stomatognathic warning signs. The cardinal physical flags include: (a) smooth, cupped erosion on the palatal surfaces of maxillary anterior teeth with ‘raised’ amalgam restorations (perimolysis); (b) bilateral, non-inflammatory enlargement of the parotid glands (sialadenosis); (c) unexplained generalized dentinal hypersensitivity in adolescent patients; and (d) erythema or traumatic petechiae on the soft palate and pharynx resulting from digital purge induction [2,8,26,41,43]. However, just as erosive tooth wear is etiologically multifactorial, salivary hypofunction and marginal gingivitis are not pathognomonic: salivary reduction may arise from medication side effects, dehydration, or mouth breathing, while gingival bleeding predominantly reflects plaque biofilm stagnation rather than progressive periodontal attachment loss. None of these physical findings can independently establish a psychiatric diagnosis. Therefore, dental practitioners must approach suspected adolescents with empathetic, non-judgmental, and confidential communication, framing observations strictly around oral tissue protection while facilitating prompt, coordinated referral to pediatricians, adolescent medicine specialists, and mental health professionals [2,7,8].

### 4.5. Strengths and Limitations

This systematic review presents several methodological strengths, including an a priori registered PROSPERO protocol, adherence to PRISMA 2020 guidelines [13], comprehensive searches up to July 2026, and an explicit stratified meta-analytic framework separating clinically diagnosed EDs from screening-positive at-risk cohorts. However, several important limitations must be acknowledged: (1) exposure heterogeneity: pooling inpatient AN cohorts with school-screened at-risk adolescents introduces clinical indirectness, which was addressed through subgroup stratification but reflects limitations in the primary literature; (2) study designs: included studies were predominantly cross-sectional or observational case–control, precluding causal inference; (3) single-center salivary evidence: both resting salivary studies originated from the same research program in Poznan, Poland, limiting generalizability; (4) small sample sizes: several studies featured small cohorts (e.g., Brandt et al., n = 12 cases), resulting in wide confidence intervals and statistical fragility; (5) mixed dentition: participants aged 9 to 15 years may present transitional dentition, potentially affecting cumulative DMFT indices; (6) unretrieved literature: eight full-text articles (19% of sought reports) could not be retrieved despite interlibrary loan requests, representing a potential source of availability bias (these comprised regional conference abstracts and non-digitized European journal articles published between 1982 and 2004 in German, French, and Italian); (7) impossibility of statistical publication bias assessment due to fewer than 10 studies per outcome; and (8) literature gaps: a complete absence of controlled studies evaluating clinically confirmed Bulimia Nervosa in strictly pediatric populations, with existing clinical evidence heavily skewed toward specialized inpatient anorexia nervosa cohorts.

## 5. Conclusions

In children and adolescents, stomatognathic manifestations associated with eating disorders vary across clinically diagnosed and screening-positive populations. Clinically diagnosed anorexia nervosa was associated with erosive tooth wear and reduced unstimulated salivary flow, whereas screening-positive adolescents also showed an association with erosive tooth wear but no statistically significant increase in caries experience. These findings should be interpreted independently, as no formal test of between-subgroup differences was performed. The certainty of evidence was very low for the main quantitative outcomes, reflecting the limited number of contributing studies, imprecision, residual confounding, inconsistency or indirectness, as applicable. In addition, evidence for reduced salivary flow was derived from only two studies conducted at a single specialized center, limiting generalizability. Because erosive tooth wear is multifactorial and not specific to eating disorders, oral findings should not be considered diagnostic in isolation. Nevertheless, dental practitioners may contribute to the recognition of suspicious oral manifestations and facilitate interdisciplinary referral when appropriate. Clinical management should emphasize individualized preventive and conservative care within a coordinated multidisciplinary approach.

## Figures and Tables

**Table 1:** Excluded studies and their reasons for exclusions (*n* = 22).

Reasons for Exclusion	Excluded Studies
Adult population Age (>18 years) (*n* = 13)	Milosevic et al., 1997 [16] Rytömaa et al., 1998 [17] Uhlen et al., 2014 [18] Johansson et al., 2012 [19] Scheutzel, 1996 [20] Touyz et al., 1993 [21] Lourenço et al., 2018 [22] Dynesen et al., 2018 [23] Valena and Young, 2002 [24] Little, 2002 [25] Ashcroft and Milosevic, 2007 [26] Johansson et al., 2020 [27] Uhlen et al., 2016 [28]
Systematic review/Meta-analysis/Review without primary empirical pediatric data (*n* = 5)	Schlueter et al., 2025 [29] Kisely et al., 2015 [30] Brown and Bonifazi, 1993 [31] Valeriani et al., 2024 [9] Chan et al., 2024 [10]
Single case report without comparative or series data (*n* = 2)	De Moor, 2004 [32] Silverstein et al., 2019 [33]
Lack of objective stomatognathic clinical outcomes (*n* = 2)	Le Grange et al., 2012 [34] Herpertz-Dahlmann, 2015 [12]

**Table 2:** Combined methodological characteristics, diagnostic classification, raw quantitative meta-analytic data, and main findings of included studies.

Author and Year	Design and Population	Diagnostic/Screening Cohort	Quantitative Raw Data	Clinical Findings and Interpretation
Hermont et al. 2013 [35]	Case–Control (15–18 y)	Severe Bulimic Risk (BITE) ED: N = 20, Ctrl: N = 80	Erosion: ED 9/20 (45.0%) vs. Ctrl 7/80 (8.8%) Raw OR = 8.53 (95% CI: 2.64–27.59) Adjusted OR = 10.04 (95% CI: 2.44–41.34) Caries: ED 16/20 (80.0%) vs. Ctrl 41/80 (51.3%)	Statistically significant elevation in tooth erosion odds among adolescents with severe bulimic risk behavior (adjusted OR = 10.04). Dental caries prevalence showed no significant difference between groups.
Hermont et al. 2021 [36]	Single-arm Cross-Sectional Case Series (15–18 y)	Bulimic Symptoms (BITE) ED: N = 62, Ctrl: N = 0	ETW Events: Mild 1/17 (5.9%), Mod 2/25 (8.0%), Severe 9/20 (45.0%) Total ETW: 12/62 (19.4%)	Descriptive observational trend between bulimic symptom severity and ETW prevalence; single-cohort evaluation without external healthy control group. Did not contribute to comparative meta-analyses.
Gokkaya and Kargul, 2022 [37]	Cross-Sectional (9–15 y)	High ED Risk (EAT-26) ED: N = 37, Ctrl: N = 75	DMFT Mean ± SD: ED: 4.12 ± 2.85 Control: 2.34 ± 1.92	Statistically significant elevation in DMFT index in high ED risk adolescents (*p* < 0.01); observational cross-sectional association, non-causal.
Paszynska et al., 2015 [14]	Controlled Clinical Comparative Study (Mean 14 y)	Diagnosed AN, Restricting Subtype (AN-R) (DSM-IV) ED: N = 28, Ctrl: N = 38	Unstimulated Flow: ED 0.28 ± 0.14 vs. Ctrl 0.45 ± 0.18 Stimulated Flow: ED 0.82 ± 0.35 vs. Ctrl 1.42 ± 0.48 Collagenase: ED 14.8 ± 4.2 vs. Ctrl 9.2 ± 3.1	Significant reduction in resting and stimulated whole salivary flow in acute AN-R inpatients. Elevated collagenase activity reflects metabolic adaptation to starvation in restrictive anorexia.
Paszynska et al., 2017 [6]	Case–Control (11–18 y)	Diagnosed AN-R (DSM-5) ED: N = 20, Ctrl: N = 21	Stimulated Parotid Flow: ED 0.21 ± 0.11 vs. Ctrl 0.48 ± 0.19 Lysozyme: ED 12.4 ± 3.8 vs. Ctrl 22.6 ± 5.4	Marked parotid flow rate reduction in restrictive AN inpatients. Significant drop in parotid lysozyme output observed.
Paszynska et al., 2018 [15]	Case–Control (Mean 15.3 y)	Diagnosed AN, Restricting Subtype (AN-R) (DSM-IV) ED: N = 30, Ctrl: N = 60	Unstimulated Flow: ED 0.25 ± 0.12 vs. Ctrl 0.46 ± 0.16 TAS: ED 0.32 ± 0.09 vs. Ctrl 0.58 ± 0.14 Vaspin: ED 1.85 ± 0.42 vs. Ctrl 0.92 ± 0.25	Significant decrease in resting whole saliva and Total Antioxidant Status. Elevated salivary vaspin levels noted as potential metabolic marker in starvation in restrictive anorexia.
Paszynska et al., 2022 [38]	Case–Control (Mean 14.9 y)	Diagnosed AN, Restricting Subtype (AN-R) (DSM-5) ED: N = 117, Ctrl: N = 103	ETW Events (BEWE ≥ 2): ED 22/117 (18.8%) vs. Ctrl 3/103 (2.9%); Raw OR = 7.72 (95% CI: 2.24–26.64) DMFT Mean ± SD: ED 3.80 ± 4.50 vs. Ctrl 1.90 ± 2.10 (*p* = 0.005) BOP (% sites): ED 20.0 ± 20.1% vs. Ctrl 3.9 ± 8.1% (*p* < 0.001) PCR (% sites): ED 43.8 ± 23.4% vs. Ctrl 13.7 ± 15.4% (*p* < 0.001)	Significantly higher ETW prevalence (OR = 7.72) and DMFT index (MD = +1.90) in diagnosed AN inpatients compared to healthy matched controls. Elevated plaque accumulation (PCR) and gingival bleeding (BOP) observed.
Jung and Jun, 2022 [39]	Nationwide Web Survey (KYRBS) (12–18 y)	Unhealthy Weight Control Behavior Cases: N = 14,210, Controls: N = 31,936	Poor Subjective Oral Health: adjusted OR = 0.89 (95% CI: 0.71–1.12, *p* = 0.32, non-significant) Self-reported toothache: OR = 1.14 (95% CI: 0.96–1.36) Self-reported bleeding: OR = 0.93 (95% CI: 0.75–1.15)	Adolescents practicing unhealthy weight control methods exhibited no statistically significant difference in poor subjective oral health. Subjective self-assessment questionnaires do not reliably substitute for clinical dental examinations.
Brandt et al., 2017 [40]	Case–Control (12–18 y)	High-Severity Bulimic Risk (BITE) ED: N = 12, Ctrl: N = 48	Erosion: ED 2/12 (16.7%) vs. Ctrl 4/48 (8.3%); OR = 2.20 (95% CI: 0.35–13.73), *p* = 0.590 Caries: ED 9/12 (75.0%) vs. Ctrl 27/48 (56.2%); OR = 2.33 (95% CI: 0.56–9.70), *p* = 0.329 DMFT: ED 1.83 ± 1.94 vs. Ctrl 1.92 ± 2.15	High-severity risk behavior was not significantly associated with dental erosion (*p* = 0.590) or dental caries (*p* = 0.329). Small case sample size (N = 12) limits statistical power.
Montecchi et al., 2003 [41]	Case Series (Descriptive) (9–18 y)	Diagnosed Anorexia Nervosa (AN-R: n = 49, AN-B/P: n = 31) (DSM-IV) ED: N = 80, Ctrl: N = 0	Erosion: 21/80 (26.3% based on Table 1 lesion tally; 20/80 [25.0%] reported as 1/4 of patients) Dentine Hypersensitivity: 24/80 (30.0%) Xerostomia: 29/80 (36.3%) Amalgam Extrusion: 11/80 (13.8%)	Descriptive clinical findings in hospitalized adolescent anorexia nervosa (49 restrictive, 31 binge-eating/purging) without healthy control group (N = 0 controls). Did not contribute to comparative meta-analyses.
Ximenes et al., 2010 [42]	Cross-Sectional (12–16 y)	High ED Risk (BITE/EAT) ED: N = 215, Ctrl: N = 435	ETW Events: ED 29/215 (13.5%) vs. Ctrl 26/435 (6.0%); Raw OR = 2.45 (95% CI: 1.41–4.28) Mucositis: ED 18/215 (8.4%) vs. Ctrl 12/435 (2.8%) Sialadenosis: ED 24/215 (11.2%) vs. Ctrl 14/435 (3.2%) Angular Cheilitis: ED 15/215 (7.0%) vs. Ctrl 8/435 (1.8%)	Statistically significant cross-sectional associations reported between screening-identified ED risk and oral mucositis, angular cheilitis, sialadenosis, and tooth wear; quantitative multivariable adjusted ORs were not extractable from the publication.
Lesar et al., 2022 [43]	Case Series (Descriptive) (10–18 y)	Diagnosed Eating Disorders (AN: n = 27 [54%], BN: n = 6 [12%], EDNOS: n = 17 [34%]) (DSM-5) ED: N = 50, Ctrl: N = 0	Plaque: 41/50 (82.0%) Marginal Gingivitis: 27/50 (54.0%) Caries: 23/50 (46.0%) Pharyngeal Erythema: 21/50 (42.0%) Angular Cheilitis: 9/50 (18.0%)	Descriptive single-arm observational cohort in adolescent eating disorder inpatients (AN, BN, EDNOS) without healthy control group (N = 0 controls). Did not contribute to comparative meta-analyses.

**Table 3:** Risk of bias and methodological quality appraisal of included studies using the Joanna Briggs Institute (JBI) critical appraisal tools.

Author and Year	Study Design	Participant Selection and Spectrum	Confounding and Exposure Measurement	Outcome Assessment and Overall Bias Judgment
Paszynska et al., 2022 [38]	Case–Control	Some concerns: Inpatient severe AN (BMI <15) vs. dental clinic controls; spectrum restriction.	Low: Confirmed psychiatric diagnosis; objective dental examination.	Moderate Risk of Bias: Setting divergence and potential unadjusted socioeconomic/lifestyle confounding.
Paszynska et al., 2018 [15]	Case–Control	Low: Matched controls; verified AN inpatient diagnosis.	Low: Standardized fasting salivary collection protocol.	Low Risk of Bias: Objective biochemical and volumetric laboratory analysis.
Paszynska et al., 2017 [6]	Case–Control	Low: Matched controls; verified AN restrictive subtype.	Low: Curby cup parotid collection; objective protocol.	Low Risk of Bias: Blinded laboratory biochemical assays.
Paszynska et al., 2015 [14]	Controlled Clinical Comparative	Low: Hospitalized AN vs. age/sex matched controls.	Low: Objective clinical diagnostic criteria.	Low Risk of Bias: Objective salivary volume and enzymatic assays; minor lack of assessor blinding.
Hermont et al., 2013 [35]	Case–Control	Low: Nested case–control from representative school sample.	Some concerns: BITE psychometric screening; multivariable regression partially adjusted for diet and brushing.	Low Risk of Bias: Validated calibration of tooth wear examiners (Kappa > 0.80).
Gokkaya and Kargul, 2022 [37]	Cross-Sectional	Low: Representative school sample aged 9–15 years.	High concerns: Lack of adjustment for dietary sugar intake, fluoride exposure, or transitional dentition.	Moderate Risk of Bias: Unadjusted confounding compromises caries outcome validity.
Hermont et al., 2021 [36]	Single-arm Cross-Sectional Case Series	Moderate concerns: Single-arm adolescent cohort identified via BITE screening; convenience school sample.	Low: Standardized BITE criteria and calibrated clinical examination.	Moderate Risk of Bias: Non-comparative descriptive design; exposure defined by psychometric screening rather than psychiatric diagnosis.
Brandt et al., 2017 [40]	Case–Control	Some concerns: Very small case sample size (N = 12 severe risk).	Some concerns: BITE screening; lack of adjustment for socioeconomic status and diet.	Moderate Risk of Bias: Low statistical power and residual confounding.
Ximenes et al., 2010 [42]	Cross-Sectional	Low: Representative adolescent school sample.	Some concerns: Self-report EAT-26/BITE screening; unadjusted for dietary acidic intake.	Moderate Risk of Bias: Cross-sectional design with potential exposure misclassification.
Jung and Jun, 2022 [39]	Cross-Sectional Survey	Low: Large nationwide representative sample (N = 46,146).	Some concerns: Web-based survey of weight loss practices without formal clinical ED diagnosis.	High Risk of Bias: Absence of clinical dental examination; outcome based strictly on self-reported perception.
Montecchi et al., 2003 [41]	Case Series	Low: Standardized psychiatric admission and DSM-IV diagnostic criteria.	Moderate concerns: Retrospective chart review; incomplete baseline confounding reporting.	Moderate Risk of Bias: Single-arm descriptive clinical case series meeting applicable JBI domain criteria.
Lesar et al., 2022 [43]	Case Series	Low: Consecutive hospital admissions with confirmed DSM-5 diagnoses.	Moderate concerns: Cross-sectional clinical evaluation; lack of longitudinal monitoring.	Moderate Risk of Bias: Single-arm descriptive clinical case series meeting applicable JBI domain criteria.

**Table 4:** GRADE Summary of Findings and Evidence Architecture Across Pediatric Eating Disorder Phenotypes.

Outcome	Contributing Studies by Subgroup	Risk of Bias	Inconsistency	Indirectness	Imprecision	Publication Bias	Pooled Effect Estimate	Overall Certainty (GRADE)
Erosive Tooth Wear–Clinically Diagnosed AN	1 study; N = 220 (Paszynska 2022)	Not serious	Not assessable (single study)	Not serious	Serious (single study; wide CI)	Not assessable	OR = 7.72 (95% CI 2.24–26.64)	VERY LOW ⊕◯◯◯
Erosive Tooth Wear–Screening-Positive/At-Risk	3 studies; N = 810 (Hermont 2013; Brandt 2017; Ximenes 2010)	Serious (residual confounding in observational studies)	Not serious (I^2^ = 46.7%)	Serious (screening-defined rather than clinically confirmed ED exposure)	Serious (few studies; wide pooled CI)	Not assessable (k = 3)	Pooled OR = 3.50 (95% CI 1.49–8.23)	VERY LOW ⊕◯◯◯
Dental Caries Experience–Clinically Diagnosed AN	1 study; N = 220 (Paszynska 2022)	Not serious	Not assessable (single study)	Not serious	Serious (single-study evidence)	Not assessable	MD = +1.90 (95% CI 0.99–2.81)	VERY LOW ⊕◯◯◯
Dental Caries Experience–Screening-Positive/At-Risk	2 studies; N = 172 (Gokkaya 2022; Brandt 2017)	Serious (residual confounding)	Serious (I^2^ = 86.8%)	Serious (screening-defined rather than clinically confirmed ED exposure)	Serious (few studies; CI crosses null)	Not assessable (k = 2)	Pooled MD = +0.88 (95% CI −0.95 to 2.71)	VERY LOW ⊕◯◯◯
Unstimulated Whole Salivary Flow–Clinically Diagnosed AN	2 studies; N = 156 (Paszynska 2015; Paszynska 2018)	Not serious	Not serious † (I^2^ = 0.0%)	Serious (both studies from the same specialized center)	Very serious (small N; k = 2)	Not assessable (k = 2)	Pooled MD = −0.20 mL/min (95% CI −0.24 to −0.15)	VERY LOW ⊕◯◯◯
Subjective Oral Health–Screening-Positive/At-Risk	1 study (Jung and Jun 2022)	Serious (self-reported outcome; no direct clinical examination)	Not assessable (single study)	Serious (screening-defined exposure and subjective outcome)	Serious (single-study evidence)	Not assessable	OR = 0.89 (95% CI 0.71–1.12)	VERY LOW ⊕◯◯◯

† The I^2^ = 0.0% estimate for salivary flow should be interpreted cautiously because only two studies contributed to the analysis, providing very limited power to detect between-study heterogeneity.

## Data Availability

The original contributions presented in this study are included in the article. Further inquiries can be directed to the corresponding authors.

## References

[1] Hornberger L.L., Lane M.A. (2021). Identification and management of eating disorders in children and adolescents. Pediatrics.

[2] Pawlaczyk-Kamieńska T., Osińska A., Śniatała R. (2016). Dental implications of eating disorders. Dent. Med. Probl..

[3] Lo Russo L., Campisi G., Di Fede O., Di Liberto C., Panzarella V., Lo Muzio L. (2008). Oral manifestations of eating disorders: A critical review. Oral Dis..

[4] Nijakowski K., Jankowski J., Gruszczyński D., Surdacka A. (2023). Eating Disorders and Dental Erosion: A Systematic Review. J. Clin. Med..

[5] Rangé H., Colon P., Godart N., Kapila Y., Bouchard P. (2021). Eating disorders through the periodontal lens. Periodontology 2000.

[6] Paszynska E., Slopien A., Dmitrzak-Weglarz M., Hannig C. (2017). Enzyme activities in parotid saliva of patients with the restrictive type of anorexia nervosa. Arch. Oral Biol..

[7] Cuadrado-Ríos S., Huamán-Garaicoa F., Cruz-Moreira K. (2023). Anorexia and bulimia nervosa in the practice of the paediatric dentist. Eur. Eat. Disord. Rev..

[8] Smorthit K., Sawbridge D., Fitzgerald R. (2021). Eating disorders and the orthodontist: Diagnosis, considerations and referral. J. Orthod..

[9] Valeriani L., Bencivenga L., Corridore D., Bossù M., Polimeni A., Vozza I. (2024). Oro-dental manifestations of eating disorders: A systematic review. J. Eat. Disord..

[10] Chan C.C.K., Nangle M.R., Dorney E., Seow W.K. (2024). Oral health status of individuals with eating disorders: Systematic review and meta-analysis. J. Dent..

[11] Chiba F.Y., Chiba E.K., Moimaz S.A.S., Matsushita D.H., Garbin A.J.Í., Garbin C.A.S. (2022). Malocclusion and its relationship with oral health-related quality of life in patients with eating disorders. Dent. Press J. Orthod..

[12] Herpertz-Dahlmann B. (2015). Adolescent eating disorders: Update on definitions, symptomatology, epidemiology, and comorbidity. Child Adolesc. Psychiatr. Clin..

[13] Page M.J., McKenzie J.E., Bossuyt P.M., Boutron I., Hoffmann T.C., Mulrow C.D., Shamseer M., Tetzlaff J.M., Akl E.A., Brennan S.E. (2021). The PRISMA 2020 statement: An updated guideline for reporting systematic reviews. BMJ.

[14] Paszynska E., Schlueter N., Slopien A., Dmitrzak-Weglarz M., Dyszkiewicz-Konwinska M., Hannig C. (2015). Salivary enzyme activity in anorexic persons—A controlled clinical trial. Clin. Oral Investig..

[15] Paszynska E., Tyszkiewicz-Nwafor M., Slopien A., Dmitrzak-Weglarz M., Dutkiewicz A., Grzelak T. (2018). Study of salivary and serum vaspin and total antioxidants in anorexia nervosa. Clin. Oral Investig..

[16] Milosevic A., Brodie D.A., Slade P.D. (1997). Dental erosion, oral hygiene, and nutrition in eating disorders. Int. J. Eat. Disord..

[17] Rytömaa I., Järvinen V., Kanerva R., Heinonen O.P. (1998). Bulimia and tooth erosion. Acta Odontol. Scand..

[18] Uhlen M.M., Tveit A.B., Stenhagen K.R., Mulic A. (2014). Self-induced vomiting and dental erosion-a clinical study. BMC Oral Health.

[19] Johansson A.K., Norring C., Unell L., Johansson A. (2012). Eating disorders and oral health: A matched case-control study. Eur. J. Oral Sci..

[20] Scheutzel P. (1996). Etiology of dental erosion-intrinsic factors. Eur. J. Oral Sci..

[21] Touyz S.W., Liew V.P., Tseng P., Frisken K., Williams H., Beumont P.J. (1993). Oral and dental complications in dieting disorders. Int. J. Eat. Disord..

[22] Lourenço M., Azevedo Á., Brandão I., Gomes P.S. (2018). Orofacial manifestations in outpatients with anorexia nervosa and bulimia nervosa focusing on the vomiting behavior. Clin. Oral Investig..

[23] Dynesen A.W., Gehrt C.A., Klinker S.E., Christensen L.B. (2018). Eating disorders: Experiences of and attitudes toward oral health and oral health behavior. Eur. J. Oral Sci..

[24] Valena V., Young W.G. (2002). Dental erosion patterns from intrinsic acid regurgitation and vomiting. Aust. Dent. J..

[25] Little J.W. (2002). Eating disorders: Dental implications. Oral Surg. Oral Med. Oral Pathol. Oral Radiol. Endod..

[26] Ashcroft A., Milosevic A. (2007). The eating disorders: 2. Behavioural and dental management. Dent. Update.

[27] Johansson A.K., Norring C., Unell L., Johansson A. (2020). Diet and behavioral habits related to oral health in eating disorder patients: A matched case-control study. J. Eat. Disord..

[28] Uhlen M.M., Mulic A., Holme B., Tveit A.B., Stenhagen K.R. (2016). The Susceptibility to Dental Erosion Differs Among Individuals. Caries Res..

[29] Schlueter N., Mulic A., Luka B. (2025). Global Prevalence of Erosive Tooth Wear. Monogr. Oral Sci..

[30] Kisely S., Baghaie H., Lalloo R., Johnson N.W. (2015). Association between poor oral health and eating disorders: Systematic review and meta-analysis. Br. J. Psychiatry.

[31] Brown S., Bonifazi D.Z. (1993). An overview of anorexia and bulimia nervosa, and the impact of eating disorders on the oral cavity. Compendium.

[32] de Moor R.J. (2004). Eating disorder-induced dental complications: A case report. J. Oral Rehabil..

[33] Silverstein L.S., Haggerty C., Sams L., Phillips C., Roberts M.W. (2019). Impact of an oral health education intervention among a group of patients with eating disorders (anorexia nervosa and bulimia nervosa). J. Eat. Disord..

[34] Le Grange D., Swanson S.A., Crow S.J., Merikangas K.R. (2012). Eating disorder not otherwise specified presentation in the US population. Int. J. Eat. Disord..

[35] Hermont A.P., Pordeus I.A., Paiva S.M., Abreu M.H.N.G., Auad S.M. (2013). Eating disorder risk behavior and dental implications among adolescents. Int. J. Eat. Disord..

[36] Hermont A.P., Pordeus I.A., Ramos-Jorge J., Paiva S.M., Auad S.M. (2021). Acidic food choice among adolescents with bulimic symptomatology: A major risk factor for erosive tooth wear?. Eat. Weight Disord.-Stud. Anorex. Bulim. Obes..

[37] Gokkaya B., Kargul B. (2022). Assessment of the eating disorders and relationship with dental caries, age, gender, and body mass index in a sample of Turkish adolescents aged 9 through 15. Niger. J. Clin. Pract..

[38] Paszynska E., Hernik A., Slopien A., Roszak M., Jowik K., Dmitrzak-Weglarz M., Tyszkiewicz-Nwafor M. (2022). Risk of Dental Caries and Erosive Tooth Wear in 117 Children and Adolescents’ Anorexia Nervosa Population-A Case-Control Study. Front. Psychiatry.

[39] Jung E.H., Jun M.K. (2022). Relationship between Risk Factors Related to Eating Disorders and Subjective Health and Oral Health. Children.

[40] Brandt L.M.T., Fernandes L.H.F., Aragão A.S., Aguiar Y.P.C., Auad S.M., de Castro R.D., Cavalcanti S.D.L.B., Cavalcanti A.L. (2017). Relationship Between Risk Behavior for Eating Disorders and Dental Caries and Dental Erosion. Sci. World J..

[41] Montecchi P.P., Custureri V., Polimeni A., Cordaro M., Costa L., Marinucci S., Montecchi F. (2003). Oral manifestations in a group of young patients with anorexia nervosa. Eat. Weight Disord.-Stud. Anorex. Bulim. Obes..

[42] Ximenes R., Couto G., Sougey E. (2010). Eating disorders in adolescents and their repercussions in oral health. Int. J. Eat. Disord..

[43] Lesar T., Vidović Juras D., Tomić M., Čimić S., Kraljević Šimunković S. (2022). Oral Changes in Pediatric Patients with Eating Disorders. Acta Clin. Croat..

[44] Lussi A., Carvalho T.S. (2014). Erosive tooth wear: A multifactorial condition of growing concern and increasing knowledge. Monogr. Oral Sci..

[45] Bartlett D.W., Lussi A., West N.X., Bouchard P., Sanz M., Bourgeois D. (2013). Prevalence of tooth wear on buccal and lingual surfaces and possible risk factors in young European adults. J. Dent..

